# Women’s perspectives on the measures that need to be taken to increase the use of health-care facility delivery service among slums women, Addis Ababa, Ethiopia: a qualitative study

**DOI:** 10.1186/s12978-021-01221-9

**Published:** 2021-08-23

**Authors:** Endalew G. Sendo, Motshedisi E. Chauke, M. Ganga-Limando

**Affiliations:** 1 College of Health Sciences, School of Nursing and Midwifery, Addis Ababa University, P.O. Box 1176, Addis Ababa, Ethiopia; 2 Department of Health Studies, University of South Africa, Pretoria, South Africa

**Keywords:** Health facility-delivery, Focused antenatal care, Slum residents, Ethiopia

## Abstract

**Background:**

Global strategies to target high maternal mortality ratios are focused on providing skilled attendance at delivery along with access to emergency obstetric care. Research that examines strategies to increase facility-based skilled birth attendance among slum residents in Addis Ababa, Ethiopia, is limited.

**Objective:**

The study aimed to explore women's perspectives on the measures that need to be taken to increase the use of the facility—delivery service among slums women, Addis Ababa, Ethiopia

**Methods:**

Qualitative exploratory and descriptive research designs were used. Participants in the study were women in the reproductive age group (18–49 years of age) living in the slum areas of Addis Ababa, Ethiopia. A purposive sampling strategy was used to select study participants. Potential participants' names were gathered from health facilities and followed to their homes for the study. Four audio-recorded focus group discussions [FGDs] were conducted with 32 participants from the three public health centers and one district hospital. The number of participants in FGDs was between 6 and 10 women. Data were analyzed simultaneously with data collection. Thematic analysis was used in data analysis, which entails three interconnected stages: data reduction, data display, and data conclusion. In addition, thematic analysis entailed evaluating the structure and content of textual data, identifying data themes, coding the themes, and then interpreting the structure and content of the themes. A codebook was first devised, discussed, and adopted by the writers before they could use this technique. Using the codebook, the theme codes were then manually produced. To explain the study results, verbatim excerpts from participants were given. The researcher used Techs' eight steps of qualitative data analysis method for analyzing the data. A multi-level life-course framework of facility-based delivery in low- and middle-income countries (LMICs) developed by Bohren et al. was used to frame the current study and link the findings of the study to the body of knowledge.

**Results:**

The FGDs included a total of 32 participants. The mean age of the overall sample was 32.6 years (± SD = 5.2). Participants' educational characteristics indicate that the majority (24 out of 32) was found to have no formal education, and two-thirds of participants were found to have one to five children. Three-fourths of them attended the ANC twice and they all gave birth to their last child at home. Two themes emerged from the analysis of focus group data, namely provision of quality, respectful and dignified midwifery care, and lack of awareness about facility delivery. These themes were described as a rich and comprehensive account of the views and suggestions made by focused antenatal care [FANC] participants on measures required to improve the use of the facility-delivery services. The findings of the study raise concerns about the effectiveness of FANC in encouraging facility-deliveries since FANC participants had not used health facilities for their last childbirth. According to the findings of the focus groups, women who took part in this study identified measures required to increase the use of health facility-delivery services among FANC participants in Addis Ababa's slum residents. It is to be expected that diligent counseling during antenatal care about birth plans would facilitate prompt arrival at facilities consistent with the desires of women.

**Supplementary Information:**

The online version contains supplementary material available at 10.1186/s12978-021-01221-9.

## Plain language summary

Global strategies for targeting high maternal mortality levels are based on ensuring eligible delivery attendance along with access to obstetric emergency care. Research examining strategies to increase facility-based skilled birth attendance among slum residents in Addis Ababa, Ethiopia, is minimal. The study aimed to examine women's perspectives on measures needed to improve the use of health facility-based delivery services.

A qualitative research design, both exploratory and descriptive, was used. Women in the reproductive age group (18–49 years of age) living in the slum areas of Addis Ababa, Ethiopia, participated in the study. The study used a purposeful sampling method. Four audio-recorded focus group discussions [FGDs] were conducted with 32 participants. In FGDs, the number of participants was between 6 and 10 women. Data were analyzed in conjunction with data collection. For the research, thematic analysis was performed.

Two issues arose from the review of data from focus group interviews, namely quality care provision, respectful and dignified midwifery care, and lack of awareness about facility delivery. These themes were identified as a detailed and comprehensive account of the views and suggestions of focused antenatal care [FANC] participants on steps needed to enhance the use of health facility-based delivery services. According to the results of the focus groups, among FANC participants in the slum residents of Addis Ababa, women who took part in this study identified steps needed to increase the use of health facility-based delivery services. It is to be expected that diligent counseling during antenatal care about birth plans would facilitate prompt arrival at facilities consistent with the desires of women.

**Supplementary Information:**

The online version contains supplementary material available at 10.1186/s12978-021-01221-9.

## Background

Pregnancy and childbirth-related maternal mortality remain a global public health issue, although it decreased by 44% from 385 deaths per 100,000 in 1990 to 216 per 100,000 live births in 2015 [1]. Sub-Saharan Africa (SSA) accounts for 66% of the world's annual maternal deaths. In developed regions, the 2015 regional maternal mortality ratio [MMR] ranged from 12 deaths per 100,000 live births to 546 deaths per 100,000 in sub-Saharan Africa [1]. The MMR remains high in Ethiopia with 412 for every 100,000 live births in 2016[2]. The Federal Democratic Republic of Ethiopia (FDRE) [3] expects to decrease maternal mortality to 199 for every 100,000 live births in 2020 and 70 or less by 2030 per the objective set by the World Health Organization (WHO). The objective set by WHO is feasible because the greater part of maternal deaths are preventable if admittance to Antenatal Care (ANC) in pregnancy, skilled care during delivery, and care and support in the weeks after childbirth were expanded [2, 4].

The proportion of Ethiopian women who received ANC from a professional provider was 62% while institutional delivery was 28% in 2016, according to the Central Statistics Agency [CSA] [2]. The low use of health facility-based delivery services seems to be one of the reasons for the slow rate at which MMR dropped during the 5 years period 2011–2016. There is a need to accelerate the drop in unskilled home deliveries and surge in facility-based deliveries to achieve sustainable development goal 3.1 [4]. Key measures to enhance maternal health are the availability of a qualified birth attendant (SBA) during childbirth, readily available emergency services, and reliable communication and referral systems [5]. There may be an unduly higher risk of maternal mortality among women living in the slums of Addis Ababa, the capital city of Ethiopia [6]. The urban slums are a particularly vulnerable community, with inadequate housing, a lack of essential utilities, and low usage of skilled care during childbirth. Despite their accessibility to health facilities, some socioeconomic constraints hinder their ability to seek necessary healthcare. There is, however, no literature on facility deliveries among women in the slums of Addis Ababa, Ethiopia. Therefore, this study aimed to explore the perspectives of focused antenatal care [FANC] participants on the measures needed to boost the use of delivery services among slum women in Addis Ababa, Ethiopia.

## Methods

### Research design

To address the purpose of this study, a qualitative, exploratory, and descriptive research design was used. In this study, the researcher could only understand the participants’ perceptions of facility-delivery and home delivery as well as their actions (non-utilization of health facility-delivery) from the participants' perspective, stated in their own words and in the context in which they lived. The primary purpose of the study was not to generalize the outcome to other settings, as it was specific to its context.

### Study setting

The current research was done at public health facilities in Addis Ababa, Ethiopia, between February and April 2018. Three health centers and one district hospital were purposively selected for the study. The public health facilities were selected because they attended to a high number of 133 women who attended FANC but attended less skilled deliveries in the past year preceding the study. The health facilities of Addis Ababa include 12 public hospitals (specialized, referral, and general), 86 public health centers, and about 720 private and non-governmental (NGO) health facilities at different levels [3].

A slum household is described in this study as a community of people living under the same roof who lack one or more of the following conditions: access to improved water, living on small business/daily labor, access to improved sanitation, adequate living space, and durability of housing. The study included Ketchne and Kolfe Keraniyo slum dwellers, who are primarily low-income residential residents [6].

### Participants and sampling method

The participants in the study included women in the reproductive age group (18–49 years of age) living in the slum areas of Addis Ababa, the capital of Ethiopia.

The women who provide rich information that appropriately answers the research questions were purposefully chosen. The women who met the requirements for eligibility have been contacted. Potential participants' names were gathered from healthcare facilities and followed to their homes for the study. The researcher ensured that the necessary information about the interviews was provided to all women who agreed to take part in the interviews and that they were followed into the communities where the health facilities are located. Participants had to be women who attended FANC in selected health facilities and gave birth to babies at home in the previous year of data collection, interact well in Amharic (local language), reside in Addis Ababa for at least 6 months, and in the reproductive age group (18 to 49 years) to be included in the sample. Exclusion criteria comprised women who attended FANC but had not experienced home delivery.

### Data collection

Focus group discussions (FGDs) were conducted by the leading author with the qualified female research assistant to address the purpose of the study, namely to gain insight into the views and perceptions of FANC participants on measures needed to increase the use of health facility-based delivery services. An interview guide was used to plan the open-ended topics in English and Amharic. The interview guide used in this study was attached as Additional file 1.

The women who met the eligibility criteria were contacted to discuss the purpose of the research, the study activities, and the request for participation in the study through the midwives/nurses in charge of the maternal and child health units of the selected hospitals and health centers.

The investigator performed FGDs with women who attended FANC and delivered live babies at home in the previous year before the study's data collection. The objective was to ask questions that elicited answers and produced maximum discussions and opinions within a given period among the study participants. Of the three selected health centers and one district hospital, four FGDs were conducted involving 32 participants. In FGDs, the number of participants was between 6 and 10 women. There were ten participants in the first FGD, eight participants in the 2nd and 3rd FGDs, and six participants in the 4th FGD.

A central topic was included in the interview guides, as well as additional questions aimed at exploring and delving deeper into various aspects of the research phenomenon. The probing questions were focused on the responses of the participants to the issue. The researcher used probing such as "please tell me more…, what do you mean by…" for questioning.

The participants were seated in a circle so that each participant had a complete and fair view of others to allow efficient contact in the FGDs. The key question for focus group discussion was: what do you think should be done to increase the use of health facility-based delivery service among FANC participants?

Additional questions included: Why do women prefer home delivery to facility-based delivery service? What are your views regarding the advantages of facility-based delivery? What were the benefits of attending antenatal care for you? What information did you receive from the health care providers about health facility-based delivery? Focus group discussions were continued until data saturation was reached, and the investigator used data saturation by group, which was the point in coding where no new codes existed in the data.

The interview process was clarified by a favourable, non-threatening, and comfortable atmosphere when the researcher introduced himself to the participants. In the private rooms of selected health facilities, the interviews took place. The FGDs were audio-recorded with the consent of the participants and notes were written during the interview to capture the original accounts of the responses of the participants and to validate their explanations by going back to the original answers. In a quiet and private space, free of distractions, and where they felt safe, the investigator conducted the interviews in Amharic. The FGDs sessions lasted approximately 60 min on average.

### Data analysis

Descriptive statistics have been used to summarize participants' socio-demographic characteristics. All FGDs were transcribed from the audio recordings and notes made during the interviews and translated into English. Data were analyzed in conjunction with data collection. The data was coded by the lead investigator. The data coding procedure was carried out according to Tesch's recommendations in Creswell [7], which included: getting a sense of the whole by reading all the transcripts carefully, select one interesting document to examine for the underlying meaning, annotate the selected document in the margin formulating topics into columns, check for any emergence of new codes and categories, combine categories that are associated to each other, shorten each category and put codes in alphabetical order, bring together the data material fitting in each category in one place and perform an initial analysis, and if necessary, recode the existing data. Using the codebook, the theme codes were then manually produced. Line-by-line coding of the various transcripts was performed. Double coding of each transcript was carried out by two of the authors. A coding comparison query was then used to compare the coding.

Thematic analysis was used in data analysis, which entails three interconnected stages: data reduction, data display, and data conclusion. In addition, thematic analysis entailed evaluating the structure and content of textual data, identifying data themes, coding the themes, and then interpreting the structure and content of the themes. A codebook was first devised, discussed, and adopted by the writers before they could use this technique. Using the codebook, the theme codes were then manually produced. To explain the study results, verbatim excerpts from participants were given.

### Trustworthiness of the study

Some procedures were employed to ensure the report's trustworthiness.

Concurrent analysis ensured that in subsequent interviews, emerging concepts were evaluated to obtain a complete understanding of the themes. To ensure the authenticity of the transcripts, interviews held in Amharic were discussed with experts in this language. Detailed field notes were maintained that allowed the results and study processes to be checked.

To ensure the study's credibility, several measures were used, including the use of the same interview guide throughout the study. For researchers to validate the methodologies used in the study, an audit trail was kept. A detailed description of the study setting, methodology (COREQ criteria were used [8]), and background of the sample was provided to ensure the transferability of the study findings to a similar context. Data were returned to participants to cross-check and validate their responses to ensure legitimacy. The researcher also received individual input from the participants on how they reacted to the data interpretation. During focus groups, the researcher spent four weeks conversing with the participants to gain a thorough grasp of their perceptions of facility-delivery service. To ensure the study's dependability, the researcher communicated with the two senior research supervisors via email, personal contact, and phone conversations frequently to track any changes made to the protocol and processes, such as reviewing, defining, and labelling themes uncovered. The themes produced were discussed by the research team to ensure that the data was complete.

To support the results, direct verbatim quotes were used and this gave voice to the women in this research. Finally, the reporting of this study adhered to the 32 criteria recommended by the consolidated criteria for reporting qualitative research [8].

### Ethics approval and consent to participate

Ethical clearance was obtained from the Research Ethics Committee of the Department of Health Studies, University of South Africa. The Addis Ababa City Government Health Bureau granted permission for the study to be carried out.

To perform the interviews, the authors received informed written consent from all participants. It underlined the voluntary nature of participation in this report. The Confidentiality of the identification and other personal details of all interviewees were ensured. The collected data were preserved electronically as audio recordings to be used as a backup format, and the transcripts and notes were stored as MS word files. To guarantee confidentiality, the MS word files were password secured.

### Conceptual model

The current study was framed and linked to the body of knowledge using a multi-level life-course framework of facility-based delivery in low- and middle-income countries (LMICs) defined by Bohren et al. [9]. The model classifies factors that affect health services utilization into five categories: individual, family, community, health-care facility, and national. This conceptual framework demonstrates how a variety of factors interact to influence the use and non-use of healthcare facilities.

## Research findings

The results of FGDs were presented below, the aim of which was to explain the views of women on measures required to increase the use of delivery services in the health facilities.

### Characteristics of participants in the study

The FGDs included a total of 32 participants. The mean age of the overall sample was 32.6 years (± SD = 5.2). Participants' educational characteristics indicate that the majority (24 out of 32) was found to have no formal education, and two-thirds of participants were found to have one to five children. Three-fourths of them attended the ANC twice and they all gave birth to their last child at home.

## Themes

Two themes emerged from the FGDs data. These themes were described as a rich and comprehensive account of the measures necessary to increase the use of facility-based delivery from the perspectives of the participants by FANC participants.

### The theme I: provision of quality, respectful and dignified midwifery care

The first theme that emerged from data analysis was the provision of quality, respectful and dignified midwifery care. Within the theme, 4 (four) sub-themes, namely perceived incompetence of staff, negative attitudes of health professionals, effective referral systems, indirect and hidden costs associated with facility delivery, and provision of adequate resources emerged. Sources of data from FGDs were as shown in Table 1.

#### Perceived incompetence of staff

The results of FGDs uncovered health care providers' perceived incompetence, lack of experience, expertise, and acceptable attitudes to care for pregnant women during pregnancy and childbirth as reasons why women do not go for delivery at the health facilities. To this effect, the participants suggested that competent staff (capable of providing quality care, characterized by respect and preservation of patients’ dignity) should be made available at the health facilities. The focus group participants suggested skills development programs which may include training, retraining, in-service education, and refresher courses to enable nurses/midwives to manage not only childbirth but also to provide respectful care to patients.

Sample responses included:

Further education and in-service training opportunities will help the staff to update their skills and knowledge to manage childbirth and provide respectful care to the women (FGD2, woman 1, 29 years).

Exploring how best midwives/nurses can be educated, developed and supported to provide high-quality midwifery care in the facilities is needed and ensuring that in-service training for staff on obstetric care is also helpful (FGD2, woman 3, 27 years).

Some of the providers indeed lack midwifery experience and skills. It seems that they weren’t trained well in school and not exposed to the clinical setting….so, the authorities should do something to improve their skills” (FGD3, woman 6, 28 years).

Besides, deploying an adequate number of supportive staff in non-clinical roles suggested freeing nurses/midwives to provide more midwifery care to minimize their work burden (FGD3, woman 1, 26 years).

#### Negative attitudes of health professionals

The study results showed that women did not prefer facility-based delivery due to the negative views of health care practitioners. According to the participants, they [providers] subject patients to mistreatment, such as verbal abuse, neglect, or denial of services. Sample responses included:

They (providers) have to respect their clients because human beings naturally need respect and dignity in childbirth (FGD 1, Woman 7, 30 years).

Indeed, staff should behave positively towards their clients and that they have to be trained ethically (FGD4, woman 3, 26 years).

There are several negligent workers. To get their support, we go there, but they talk and talk about their private problems. So, going there is not advisable or they must behave morally (FGD 1, Woman 2, 34 years).

The providers will beat you, and they will shout at you without any mistakes. So, they should first stop such abusive behaviors if they want us to go there for childbirth. I mean they need to have sound professional ethics and behavioral change (FGD2, woman 4, 33 years).

#### Effective referral systems

Patient referral is a medical judgment dependent on many variables, including the prescribing team's expertise, testing resources, the health institution's availability of specialist facilities, the level of service at the referral institution, the cost of treatment, distance, transportation, contact, patient travel, and consumer travel viability. Delays in access to referral facilities have been seen in this study as a significant contributing factor to feto-maternal deaths Sample responses include:

Due to shortages of supplies or diagnostic facilities, some women are referred from one facility to another. The comparison should be based on the woman's condition (FGD2, woman 8, 40 years).

You are referred here and there when you go there (HF), and finally end up with a dead baby. The authorities should work hard to improve this problem (FGD2, woman 3, 24 years).

It is essential to provide ambulance services with an effective referral system to district hospitals to boost facility-based delivery services because poor women cannot pay for tertiary-level hospitals (FGD3, woman 5, 29 years).

##### Indirect and hidden costs associated with facility delivery

Although maternal care is free in Ethiopia, the indirect expenses of childbirth were too high for many women who considered themselves to be too poor to give birth in a health facility. For example, economically disadvantaged women, particularly those whose families rely on sporadic labor, may have difficulty getting cash to pay for gloves, drugs, and lab testing during facility-based delivery care at the time of service. Some women considered costs other than the immediate cost of childbirth to be "unseen" and difficult to budget for. The sample response.

When you go to (HF) for labor, you have to buy gloves, drugs, and lab tests, among other things (FGD1, woman 7, 30 years)..

You need someone to get a taxi and pay for it so you can get to the hospital (FGD2, woman 6, 34 years).

#### Provision of adequate resources

The study results showed that the Ethiopian government encourages all women to deliver at the health facility. Therefore, it follows that medications and medical services such as ultrasound tests should be made available because some poor women cannot afford to pay for them when they are referred to other places for examination. Sample responses include:

At present, the government encourages all women to deliver at the health facility. So, drugs, and diagnostic facilities such as ultrasound examinations should be made accessible because some poor women can't afford to pay for it when they are referred for examination to other places (FGD1, woman 1, 26 years).

There are no or limited basic medical supplies, delivery beds and diagnostic facilities such as ultrasound exams, drugs, etc. if you visit public health facilities (FGD1, woman 5, 32 years).

There are no bed sheets, soaps, and other things and it should be worked on by the government (FGD3, woman 7, 36 years).

Due to shortages of supplies or diagnostic facilities, some women are referred from one facility to another (FGD 3, woman 2, 38 years).

### Theme II: lack of awareness about facility delivery

The second theme to emerge from data analysis was a lack of awareness about facility delivery*.* Within the theme, three subthemes ANC providers do not promote facility delivery; fear of cutting, and family support emerged. Sources of data from FGDs were as shown in Table 2.

**Table 1:** Theme I: provision of quality, respectful and dignified midwifery care

Theme	Sub-themes	Data source: FGDs
Provision of quality, respectful and dignified midwifery care	I. Perceived incompetence of staff	FGDs 1, 2, 4
	II. Negative attitudes of health professionals	
	III. Effective referral systems	FGDs 1, 2, 3, 4
	IV. Indirect and hidden costs associated with facility delivery	FGDs 1, 2, 3
	V. Provision of adequate resources	FGDs 1, 2,3

**Table 2:** Theme II: lack of awareness about facility delivery

Theme	Sub-themes	Data source: FGDs
Lack of awareness about facility delivery	I. ANC providers do not promote facility delivery	FGDs 1, 2, 3, 4
	II. Fear of cutting/unnecessary procedures	FGDs 1, 2, 3
	III. Family support during childbirth	FGDs 1, 2, 3, 4

#### ANC providers do not promote facility delivery

The results of the FGDs showed a lack of understanding among some of the women who participated in the study of the significance of facility-based births. Women were unable to access facility delivery due to a lack of planning for labor, including the decision of where to deliver, transportation preparation, and obtaining money to pay for associated childbirth fees. Women may not be getting enough information from ANC providers about the importance of receiving care in a facility during labor and delivery. Providers may also overlook the importance of planning, recommending only facility-based deliveries for women who exhibit obvious danger signs. As a result, a proposal to increase awareness through effective information sharing (between providers and women receiving care) and health education has been proposed. According to the WHO criteria for improving maternal and new-born treatment in health facilities [1], information about their care and relationships with staff should be accessed by both women and their families. Simple and precise exchange of information should occur. Sample responses include:

I did not receive any information about a facility's delivery. She (the midwife) just tested me and told me to come to the next appointment; I believe I have the right to that information (FGD 3, woman 4, 31 years).

The importance of health facility delivery must be explained to us by the ANC clinic's service providers. Nobody brought it up to us. As a result, we chose to give birth at home. (FGD 1, woman 9, 35 years).

I believe there should be a plan in place for where to give birth, transportation preparation, and related costs but we rarely do, and caregivers seemed to be inattentive in supporting us with planning during ANC (FGD 2, woman 5, 37 years).

Some women said that health facilities performed unnecessary procedures such as caesarean section and episiotomy, according to the study's findings. Women reported fear of cutting as a deterrent to using a facility to deliver their babies. Sample responses include:

Particularly in private facilities, Caesarean section delivery is quite common.... I think they just do it without sufficient medical reasons (FGD 3, woman 2, 39 years).

Several women are cut and stitched for the reasons I don’t know. (FGD 2, woman 4, 40 years).

Fear of the delivery by the Caesarean section discourages us [women] from coming for delivery based on health facilities (FGD 1, woman 2, 42 years).

#### Family support during childbirth

The majority of the women in the focus groups were concerned about health-care rules that prevent family members (including spouses) from providing the necessary physical and emotional assistance during labour and delivery. Many of them recommended allowing their family members [and husbands] to assist them in labour wards. This finding was evident in the sample responses:

My sister lives in Europe and she tells me that her husband plays a big role in helping her through labour and childbirth. But our husbands are not permitted to see their wives in the labour unit……this needs to be changed because they [husbands] should be able to be with their wives (FGD 4, woman 2, 33 years).

[…] If my husband were allowed to accompany me to the delivery room, I would be happier because he had to share my pain and misery as well. I, therefore, hope that the facility will one day recognize this problem and permit my husband to join the labour wards (FGD 2, woman 6, 34 years).

The WHO guidelines for enhancing maternal and newborn care in health facilities [1] support this finding, noting that women can choose to have a partner of their choice present for labor and childbirth and, that they must obtain support to improve capacity during delivery.

## Measures for enhancing facility-based delivery, as suggested by women

Below are the measures proposed by women to increase the use of facility delivery:

1. Provision of quality, respectful and dignified midwifery care
  *Increase the availability of qualified staff*: In this study, women, therefore, proposed steps such as examining how best midwives/nurses can be trained, developed, and assisted to provide high-quality midwifery treatment in the facilities and ensuring that frequent updates are carried out for in-service training for obstetric care staff. Furthermore, the deployment of ample numbers of supportive staff in non-clinical positions indicated free nurses/midwives to provide more midwifery care to reduce their workload.
  *Development of expertise*: Through in-service training, refresher courses, and continued education, the skills of service providers employed in public health facilities must be strengthened. The abilities described included communication, positive attitude, interpersonal skills, and empathy in particular.
  *Effective referral system:* Render prompt referrals for emergency treatment, ensure arrangement for ambulance service and care during travel to a higher-level health facility. Reduce women's referral to health facilities in the district of equal status (prevent delay in seeking care).
  *Adequate resources:* The results indicate that participants in the study considered that some of the health facility buildings were mainly small with minimal delivery beds (couches) and waiting rooms for women in labour. The women, therefore, propose that there should be more delivery beds (couches), medications, and medical services such as ultrasound testing.
2. Lack of awareness about facility delivery
  *ANC providers do not promote facility delivery.* Women may not be getting enough information from ANC providers about the importance of receiving care in a facility during labour and delivery. Providers may also overlook the importance of planning, recommending only facility-based deliveries for women who exhibit obvious danger signs. As a result, a proposal to increase awareness through effective information sharing (between providers and women receiving care) and health education has been proposed. Both women and their families should receive and communicate with staff with details about their treatment plan. Simple and precise exchange of information should occur. Improve the understanding of health professionals about pregnant women's access to information and adequate health care. Despite having easy access to health care, slum women did not give birth in health facilities. Interventions targeted at boosting awareness can help increase the number of births in health facilities among slum women.
  *Family support during childbirth:* Present opportunities for women to choose to have a companion of their choice present for labour and delivery and to receive support to enhance capacity during delivery, particularly the presence during delivery of husbands or partners.

### Personal reflections as regards focus group discussions

Through using bracketing, the researcher ensured that his beliefs, viewpoints, and experiences about the phenomena under investigation did not influence data collection and data analysis. The gender of the researcher (male nurse-midwife) and context did not influence the data collection process and data analysis for the current study in any way.

During the second focus group interview, the researcher aimed to elicit the views of the silent members of the FGD by asking them to contribute. After the interview had finished, one study participant arrived late. When the participant calmed down and resumed the interview, the researcher postponed the session. Otherwise, during the entire interview process, the study participants were cooperative and conformed to the ground rules.

## Discussion

The article examines the facility delivery of a group of women who received focused antenatal care services (FANC) at public health facilities in a slum setting in Addis Ababa. A multi-level life-course framework developed by Bohren et al. [7] for the facility-delivery in low- and middle-income countries (LMICs) was used to frame the current study and relate the study's results to the body of knowledge. Bohren et al. constructed this paradigm by investigating how early life experiences influence later decisions and actions, and how these experiences differ across individual, family, community, health facility, and national level effects, using a multi-dimensional life course approach.

The results of the FGD interviews showed that women did not prefer facility-based delivery because of health professionals' perceived incompetence and negative attitudes, as well as inadequate service at health facilities. This finding is consistent with the results of the previous study. For example, women who reported negative experiences in facilities and lacked faith in the abilities of health staff, who they perceived to be undertrained, incompetent, and inexperienced, were less likely to want delivery of facilities [7]. The results of the study showed the perceived incompetence of health care providers, lack of expertise, capacity, and acceptable attitudes to care for pregnant women during pregnancy and childbirth as reasons why women do not go for delivery at health facilities. Providers were described in this study as verbally and physically aggressive, rude, bossy, abusive, easily angered, and lack of compassion. Similar findings were reported in previous studies [9–12].

According to the participants, patients should not be subjected to bullying by professional employees, such as verbal assault, incompetence, or denial of services. To this end, the participants suggested that competent personnel (capable of providing reliable, distinguished care and safeguarding the dignity of patients) should be put at the disposal of health facilities.

Inadequate access to travel resources played a critical role in whether or not a woman in the labor was able to attend a health facility on time. Unavailability of transportation access, good roads, adequate funds, and communication networks often make it a long process to arrange referrals for obstetric problems [9, 13]. The findings show that study participants considered that some of the buildings of the health facility were mainly small with limited delivery beds (couches) and waiting rooms for women in labour. The participants ended up giving birth at home because of insufficient access to health facilities, according to the study results. The difficulty of getting transport to the health facility, in particular emergency services, especially at night, resulted in women delivering babies at home. Therefore, women recommend that more delivery beds (couches), medicines, and medical facilities, such as ultrasound exams, should be made available. The results of the research are consistent with prior studies [6, 9].

Referring a patient is a medical decision determined by many factors, including the skills of the referring staff, the tools for diagnosis, the availability of a health institution with specialist facilities, the quality of care at the referral institution, the cost of care, distance, transportation, communication, someone to travel with the patient, and feasibility of travel by the client [14, 15]. In the previous report, delays in obtaining access to referral services were seen as a major factor leading to feto-maternal deaths [6, 16]. The same authors suggest oversight of care providers and increasing transparency and suggest evaluations of referrals for obstetric emergencies to strengthen obstetric care referral processes to avoid delays.

In the study communities, home delivery was a common occurrence. The decision of women to give birth at home was influenced by a lack of knowledge about facility-based childbirth, according to the study results. Some of the participants indicated that they did not know about the facility-based delivery program at public health facilities.

The findings of the study are consistent with other slum-based studies in India [17, 18] that found because of an awareness deficit about the advantages of health facility-based childbirth, women prefer home delivery. Several researchers believe that ANC workers do not properly advise women on the importance of facility-delivery services, possibly due to heavy workload and time constraints due to intentionally complicated problems with their clients [9, 11, 19]. While it is crucial to improve awareness and assist mothers in preparing for birth, persistent efforts are required to eliminate the barriers that prevent skilled birth attendance and the utilization of a health facility for delivery.

Among some research participants, the results of the study also showed a belief that home delivery is for women with a history of normal delivery. The results of the study are consistent with Øxnevad's [20] research on attitudes and practices related to home-based delivery, and Bedford, Gandhi, Admassu, and Girth's [21], a qualitative study on the position of childbirth in rural Ethiopia. The birthing process was considered a natural occurrence, according to the results of the same research, and women considered home delivery first and considered facility-based delivery only if complications arose. Women may believe that going to ANC will lessen their chances of having a difficult delivery, and they may utilize it as a prophylactic approach to ensure a healthy pregnancy and delivery at home [9].

The results of the survey conducted by Yaya et al. [22] showed that one in four women in Ethiopia reported that delivery dependent on health facilities was not important considering that delivery is a normal occurrence and not an illness needing services from health facilities. Kebede et al. [23] performed a comprehensive analysis on factors associated with Ethiopia's institutional delivery service and found that women who faced difficulties during pregnancy were 2.8 times more likely than those who did not face problems during pregnancy to use health care facility-based delivery. The single most effective technique for avoiding maternal and neonatal deaths is delivery at a health facility, according to Kebede because it ensures careful treatment of childbirth complications and or prompt transfer to deliver women to higher levels where complications of childbirth can be better handled.

Some of the women had a belief, according to the study results, that unnecessary procedure are conducted during delivery at health facilities. This result is consistent with the multi-level life-course framework of facility-based delivery in low- and middle-income countries (LMICs) of Bohren et al. [9], which indicates that one of the reasons women prefer home to the facility-delivery could be the medicalization of childbirth. According to the model, women may fear various undesirable treatments and procedures such as episiotomies and cesarean sections in low- and middle-income countries and may prefer to deliver at home. This fear is largely focused on the belief that birth is a "normal" process that is the "natural passage rite" of a woman without any justification for delivery at a health facility [9–11].

A notable finding is that women who attended FANC did not make plans for preparedness for emergencies and complications, as planned following WHO. The WHO advises that all pregnant women prepare a written plan to deal with birth and any unforeseen adverse events that may occur during pregnancy, birth, or the immediate postnatal period, such as complications or emergencies [4]. Birth preparedness is the process of preparing for normal birth, whereas preparation for complications refers to anticipating the required steps in the event of an emergency. Emergency preparation is the mechanism by which all the measures that need to take place immediately in the case of an emergency are identified and decided, and the specifics are known by all concerned and the appropriate preparations are made. At each FANC evaluation and one month before the planned date of birth, arrangements should be discussed with the eligible attendant [17, 24].

Some of the participants indicated that the presence of partners, family members, friends, and neighbors offer the required support and assistance during delivery at home. These results were consistent with the results of previous studies of urban poor in Mumbai-India and Nigeria, where more than half of them were delivered outside hospital facilities and 81.8% of those deliveries were not attended by a trained health provider [25, 26]. Findings from this study are similar to maternal practices and behaviors in the urban slums of Bangladesh [27]. The baseline study indicated that one-quarter of newly delivered women had at least four antenatal care visits in the Slums of Bangladesh, where 85 percent of births took place at home and 58 percent of deliveries were supported by untrained traditional birth attendants.

Adinew and Assefa [28] reported similar findings that Ethiopian women involved in their study selected home-based and conventional birth attendants for facilities-based delivery and health professionals, respectively. The same authors describe that the preference was based on the familiarity, comfort, and convenience of the home setting. Despite good physical access, slum women did not give birth at health facilities. Increasing the number of births in health facilities among slum residents can be enhanced by interventions aimed at raising awareness.

The presence of a person of the woman’s choice to provide social support during childbirth has shown to have a positive effect to use a facility [29, 30]. It is necessary to inform mothers and families about the usual signs of labour in planning for normal birth, with a particular focus on what to do when labour begins, and to ensure that everyone calls the facility or another eligible attendant for birth as soon as possible. In particular, these instructions must be written in the local language. All the necessary details about safe and clean delivery must be given to the woman, but her choice of the chosen place and who she wants to be during work should be respected. The strategy should include identifying sources of support during the birth and the immediate postnatal period for her and her family. Our findings on maternal care practices are also important for the development of maternal and newborn child health services in Addis Ababa's slums. Counseling and behavioral change messaging for women should be included in interventions in urban areas, particularly in slums, to support optimal maternal care throughout pregnancy, delivery, and the postpartum period**.** These initiatives have the potential to improve maternal care practices, including facility-delivery in urban slums.

One of the reasons women choose to deliver their kids at home is the exorbitant cost of delivering in a health institution. Although maternal care is free in Ethiopia, the indirect expenses of childbirth were too high for many women who considered themselves to be too poor to give birth in a health facility. Economically disadvantaged women, particularly those whose families rely on sporadic labor, may have difficulty getting cash to pay for gloves, drugs, and lab testing during facility-based delivery care at the time of service. Some women considered costs other than the immediate cost of childbirth to be "unseen" and difficult to budget for.

A similar study in Lagos, Nigeria [31], and Addis Ababa Slums in Ethiopia [6] showed a financial barrier to women living in urban poor populations using health facilities for delivery care. Policymakers and government authorities should take note of our findings to provide skilled delivery care in neglected metropolitan areas.

## Conclusions

In summary, the facility-based delivery service is a multifaceted subject, which is characterized by different factors, including the characteristics of the pregnant woman herself, her close family circle, the community in which she lives, the local health facility, and her country's background.

The results of the study raise questions about FANC's efficacy in supporting facility-based deliveries since FANC participants had not used health facilities for their childbirth. According to the findings of the focus groups, women who took part in this study identified their opinions on measures required to increase the use of health facility-based delivery services among FANC participants in Addis Ababa's slum residents. It is to be expected that diligent counseling during antenatal care about birth plans would facilitate prompt arrival at facilities consistent with the desires of women. It is imperative to use ANC visits to alert women about birth preparedness and readiness for complications, the use of facility deliveries, and the risks of home delivery for mothers and infants. It is vital to present opportunities for women to choose to have a companion of their choice present for labor and delivery and to receive support during delivery to improve capacity, especially the presence of husbands or partners during delivery. Future research can assess whether women are satisfied with the maternal care provisions at public health facilities and whether they will return for future delivery or advise others to deliver there.

### Contribution of the study

Research examining strategies to increase facility skilled birth attendance among slum residents in Addis Ababa, Ethiopia, is minimal. The results of this study added to the existing body of knowledge among participants of antenatal care at selected public health facilities in the study setting, regarding the perspective of facility-based delivery services. In guiding health care practitioners, understanding women's perspectives on facility and home deliveries are important and helpful in designing women-centered guidelines that address adverse perceptions of health facility-delivery among slum residents, so that the number of home deliveries could decrease and the number of facility deliveries could increase among FANC participants in Slums.

### Strengths and limitations of the study

This research is one of the first studies in Ethiopia to examine access in urban slum settings to facility-based delivery care. It should also be clear that local and global works substantiate the emerging themes. The results are also important to health agencies that need to optimize delivery services based on health facilities. Our study, however, has some limitations. When evaluating the findings, some limitations must be taken into account. The results are based on self-reported maternal care practices, which may or may not reflect actual practices. Furthermore, we excluded primigravidas, therefore we were unable to capture their thoughts and perceptions. However, we are convinced that the findings represent actual practices and are indicative of urban slum maternal care practices, based on the consistency of findings in qualitative interviews.

Concerns about the small sample size, data interpretation, and bias are common criticisms of qualitative research. For the research process to be as objective as possible, the researcher was self-conscious and aware of his immersion in the research process in this study. The thorough description of the sample, data collection methods, and data analysis procedure, according to the researcher, highlighted the study's translucent character.

## Supplementary Information

**Additional file 1**. Interview Schedule for FGDS.

## Data Availability

All data generated or analyzed during this study are included in this published article and its supplementary information files.

## Abbreviations

ANC: Antenatal care
CSA: Central Statistics Agency
FANC: Focused antenatal care
FGDs: Focus group discussions
MMR: Maternal mortality ratio
SBA: Qualified birth attendant
WHO: World Health Organization
