# Supplementary material for: Women’s perspectives on the measures that need to be taken to increase the use of health-care facility delivery service among slums women, Addis Ababa, Ethiopia: a qualitative study
Source: Reprod Health. 2021 Aug 23;18:174. doi: 10.1186/s12978-021-01221-9 (PMC8381586; doi:10.1186/s12978-021-01221-9)
Supplement: Supplementary file 1 — Additional file 1. Interview Schedule for FGDS. [file 12978_2021_1221_MOESM1_ESM.docx]

**Additional file 1**

**A. Interview Schedule for FGDS (English Version)**

Thank you for making time to take part in this interview. My name is *Endalew Gemechu Sendo* and I would like to talk to you about the measures needed to enhance the utilization of facility-based delivery among attendees of FANC in Addis Ababa, Ethiopia.

Please remember that you are under no obligation to participate in this interview. You can withdraw from the study at this point or end the interview at any point during the interview without explanation or consequences. I promise to treat all information collected from this interview as highly confidential and it shall not be reported in a manner that identifies or links you with the results of the study. The interview should take about thirty (45) minutes. Even though I will be taking some notes, I cannot write fast enough that is why I will be recording this interview so I do not miss any of your comments. Because we are on tape, please make sure that you speak up so that we do not miss your important responses.

Do you have any questions regarding what I have just explained to you? I hope you have all signed the confidentiality binding form.

**Interview schedule**

The central question that was asked was “*What do you think should be done to enhance the utilization of health facility-based delivery among attendees of FANC?*

Specific group interview questions fell into the categories:

1. Discussion of participants experience of facility-based delivery services

- *Did you deliver any/ all of your babies at a health care facility?*
- *How did you experience health care facility-based delivery?*
- *Were you satisfied with the services rendered?*

1. Impressions and views of facility-based delivery services

- *What are your impressions or views of facility-based delivery?*
- *What do you think of delivering babies at the health facility?*

1. Barriers to utilization of facility-based delivery services

*What do you think are the reasons people choose to deliver their babies at home?*

*What change do you want to see in the facility- delivery services?*

1. Measures to enhance facility-based delivery services

- *What is your recommendation for improving facility-delivery services in your health facility?*
- *Please share with us your views on measures needed to enhance the utilization of health facility-based delivery services.*

**Thank you very much for taking part in this study.**

**B. የቡድን ውይይት ጥያቄዎች (Interview Schedule for FGDS) - Amharic Version**

በውይይቱ ላይ ለመሳተፍ ፍቃደኛ ስለሆኑ አመሰግናለሁ፡፡በዛሬው እለት ውይይታችንን የምመራው እኔ **እንዳለው ገመቹ ሰንዶ** በደቡብ አፍሪካ ዩኒቨርስቲ የጤና ትምህርት ክፍል የዶክትሬት ተማሪ ነኝ፡፡ ውይይታችን የሚያተኩረው የጤና አገልግሎት በጤና ማእከል ውስጥ እንዴት መሻሻል እንዳለበት ለመወያየት ነው። በዚህ የቡድን ውይይት ላይ የሚኖሮትን ተሳትፎ በማንኛውም ጊዜ ለእርስዎ ምቾት የማይሰጥ ሆኖ ሲያገኙት ማቋረጥ ይችላሉ፡፡ እኔም የግል ሚስጢራችሁን ለመጠበቅ ቃል እገባለሁ፡፡ ውይይቱ በአማካይ 45 ደቂቃ ይወስዳል፡፡ ምንም እንኳን ማስታወሻ ብይዝም ድምጻችሁን ግን በመቅረጫ እቀርጻለሁ፡፡ ምክንያቱም የሚትሰጡኝን የትኛውንም መረጃ ማጣት ስለማልፈልግ ነው፡፡

ከላይ በተደረገው ገለጻ ላይ ጥያቄ አላችሁ?

የተወሰኑ የቡድን ውይይት ጥያቄዎች እንደሚከተሉት ናቸው፦

1. **ተሳታፊዎቹ በጤና ተቋም መውለድ ላይ ያላቸው ገጠመኝ**

- በጤና ተቋም ውስጥ ወልደው ያውቃሉ?
- በጤና ተቋም መውለድ ጋር ተያይዞ ያለዎትን ገጠመኝ ያጋሩን::
- በአገልግሎቱ ረክተዋል?

**በጤና ተቋም መውለድ ጋር ተያይዞ ያለዎትን ሃሳብና አመለካከት**

- በጤና ተቋም መውለድ ጋር ተያይዞ ያለዎትን ሃሳብና አመለካከት ምን ይመስላል?
- በጤና ተቋም ውስጥ መውለድን እንዴት ያዩታል?

**በጤና ተቋም ውስጥ መውለድ ጋር ተያይዞ ያሉት ተግዳሮቶች**

- እናቶች በቤት መውለድን የሚመርጡት ለምን ይመስሎታል?
- በጤና ተቋም ውስጥ እንዲሻሻል የሚፈልጉት ነገሮች ምንድን ናቸው?

**እናቶች በጤና ተቋም ውስጥ እንዲወልዱ መወሰድ ያለባቸው እርምጃዎች**

- በጤና ተቋም ውስጥ ያሉት ክፍተቶች እንዲሻሻሉ ምን ምክረ ሃሳብ ይሰጣሉ?
- በጤና ተቋም ውስጥ ያሉት ችግሮች እንዲቀረፉ ምን እርምጃ መወሰድ አለበት ይላሉ?

**ለተሳትፎዎ እጅግ አመሰግናለሁ**
